# AAGL practice report: practice guidelines on intrauterine adhesions developed in collaboration with the European Society of Gynaecological Endoscopy (ESGE)

**DOI:** 10.1186/s10397-017-1007-3

**Published:** 2017-05-01

**Authors:** AAGL Elevating Gynecologic Surgery

**Affiliations:** Royal Hospital for Women, Sydney, Australia

## What is new in this report?

Substantial progress has been made since publishing previous practice guidelines on intrauterine adhesions (IUAs) [1]. Large-scale series, although retrospective, have reported clinical outcomes. Randomized controlled trials (RCTs) have investigated both primary and secondary adhesion prevention including solid and semi-solid barriers, although individual surgical techniques have not been rigorously studied. Recent human studies documenting successful pregnancy outcomes for bone marrow-derived stem cell (BMDSC) treatments following intermittent hysteroscopy are reported. This may provide a new avenue for research, although high quality data demonstrating efficacy are required before being introduced as a treatment option for women with symptomatic IUAs (Asherman syndrome).

In order to encourage their wide dissemination, these guidelines are freely accessible on the GYNS and JMIG websites.

## Background

Intrauterine adhesions have been recognized as a cause of secondary amenorrhea since the end of the nineteenth century [2], and in the mid-twentieth century, Asherman further described the eponymous condition occurring after pregnancy [3, 4]. The terms “Asherman syndrome” and IUAs are often used interchangeably, although the syndrome requires the constellation of signs and symptoms (in this case, pain, menstrual disturbance, and subfertility in any combination) related to the presence of IUAs [3, 4]. The presence of IUAs in the absence of symptoms may be best referred to as asymptomatic IUAs and are of questionable clinical significance. In these guidelines, we use the term “IUAs” specifying whether or not they are associated with clinical symptoms.

### Identification and assessment of evidence

The AAGL Practice Guidelines were produced by searching electronic databases including MEDLINE, PubMed, CINAHL, the Cochrane Library (including the Cochrane Database of Systematic Reviews), Current Contents, and EMBASE for all articles related to IUAs up to and including week 4 of April 2016. The MeSH (in MEDLARS) terms included all subheadings, and keywords included Asherman syndrome; Intrauterine adhesions; Intrauterine septum and synechiae; Hysteroscopic lysis of adhesions; Hysteroscopic synechiolysis; Hysteroscopy and adhesion and Obstetric outcomes following intrauterine surgery.

The search was not restricted to English language literature; committee members fluent in languages other than English reviewed relevant articles and provided the committee with relative information translated into English. All published works were included from the electronic database searches, and relevant articles not available in electronic sources (e.g., published before the beginning of electronic database commencement) were cross-referenced from hand-searched bibliographies and included in the literature review. When necessary, authors were contacted directly for clarification of published data. All studies were assessed for methodological rigor and graded according to the US Preventive Services Task Force classification system outlined in the previous practice guidelines on IUAs [1]. Recommendations were based on the best available evidence, where possible, and where such evidence was not available, upon consensus of the expert panel.

### Diagnosis

In women with suspected Asherman syndrome, physical examination frequently fails to reveal abnormalities [5, 6]. Blind, transcervical uterine sounding may reveal cervical obstruction at or near the level of the internal os [6]; however, adhesions higher in the cavity or more laterally may not be demonstrated in this manner. Hysteroscopy has been established as the criterion standard for diagnosis of IUAs [7]. Compared with radiologic investigations, and provided the endometrial cavity is accessible, hysteroscopy more accurately confirms the presence, extent, and morphological characteristics of adhesions and the quality of the endometrium. It provides a real-time view of the cavity, enabling accurate description of location and degree of adhesions, classification, and concurrent treatment of IUAs [8].

Hysterosalpingography (HSG) using contrast dye has a sensitivity of 75 to 81%, specificity of 80%, and positive predictive value of 50% compared with hysteroscopy for diagnosis of IUAs [9, 10]. The high false-positive rate (up to 39%) [11] limits its use, and it does not detect endometrial fibrosis [4] or the nature and extent of IUAs [12], and therefore, use should be confined to that of a screening test.

Sonohysterography (SHG; also called saline infusion sonography (SIS) or gel infusion sonography (GIS)) was found to be as effective as HSG, with both reported to have a sensitivity of 75% and positive predictive value of 43% for SHG or SIS/GIS and 50% for HSG, compared with hysteroscopy [10, 13]. Imaging techniques do appear to be hierarchical with two-dimensional gray-scale transvaginal ultrasonography having a sensitivity of 52% and specificity of 11% compared with hysteroscopy [13]. Three-dimensional (3D) ultrasonography may be more helpful in the evaluation of IUAs, with sensitivity reported to be 87% and specificity of 45% when compared with 3D SHG (although this study did not compare with hysteroscopy) [14]. 3D SHG has a high specificity of 87% although a lower sensitivity of 70% when compared with the standard, hysteroscopy [15].

Newer techniques currently being investigated include power Doppler sonography where studies suggest high resistance flows that are associated with poorer obstetric outcomes [16], and the addition of contrast color power angiography to 3D ultrasonography may have a role in both initial assessment and prognosis for women with Asherman syndrome [17]. Initial assessments of magnetic resonance imaging (MRI) for the diagnosis of IUAs show few advantages over less costly alternatives [18–20], with more recent assessment of gadolinium-enhanced images showing some promise [21, 22]. None of these techniques have been fully evaluated or can be recommended for routine practice until further research is undertaken [23].

#### Guidelines for diagnosis of intrauterine adhesions

1. Hysteroscopy is the most accurate method for diagnosis of IUAs and should be the investigation of choice when available. Level B.
2. If hysteroscopy is not available, HSG and SHG are reasonable alternatives. Level B.
3. Magnetic resonance imaging should not be used for diagnosis of IUAs outside of clinical research studies. Level C.

### Classification

Classification of IUAs is useful because prognosis is related to the severity of disease [8]. A number of classification systems have been proposed for IUAs, each of which includes hysteroscopy to determine the characteristics of adhesions [24]. To date, there are no data from any comparative analysis of these classification systems. Table 1 gives the available classification systems and their key features.

**Table 1:** Classification of intrauterine adhesions

Source	Summary of classification
March et al. [7]	Adhesions classified as minimal, moderate, or severe based on hysteroscopic assessment of the degree of uterine cavity involvement.
Hamou et al. [25]	Adhesions classified as isthmic, marginal, central, or severe according to hysteroscopic assessment.
Valle and Sciarra [26]	Adhesions classified as mild, moderate, or severe according to hysteroscopic assessment and extent of occlusion (partial or total) at HSG.
European Society for Hysteroscopy [27]	Complex system classifies IUAs as grades I through IV with several subtypes and incorporates a combination of hysteroscopic and HSG findings and clinical symptoms.
American Fertility Society [28]	Complex scored system of mild, moderate, or severe IUAs based on extent of endometrial cavity obliteration, appearance of adhesions, and patient menstrual characteristics based on hysteroscopic or HSG assessment.
Donnez and Nisolle [29]	Adhesions classified into six grades on the basis of location, with postoperative pregnancy rate the primary driver. Hysteroscopy or HSG are used for assessment.
Nasr et al. [30]	Complex system creates a prognostic score by incorporating menstrual and obstetric history with IUA findings at hysteroscopic assessment.

#### Guidelines for classification of intrauterine adhesions

1. Intrauterine adhesions should be classified as prognosis is correlated with severity of adhesions. Level B.
2. The various classification systems make comparison between studies difficult to interpret. This may reflect inherent deficiencies in each of the classification systems. Consequently, it is currently not possible to endorse any specific system. Level C.

### Primary prevention

There are eight RCTs reporting outcomes on methods for primary prevention of IUAs following surgical procedures [31–38]. The first RCT evaluated the value of using oral estrogen postoperatively following hysteroscopic septoplasty and reported no significant difference of de novo adhesion formation [31]. At second-look hysteroscopy, there were no adhesions in 42 women assigned to take 2 mg of estradiol valerate per day for 30 days postoperatively while synechiae were seen in 3 of 43 women (7%) in the placebo group. There was no difference in subsequent pregnancy rates (37% estrogen group vs 41% placebo group) at up to 2 years follow-up. Similar data were reported in a second RCT of 100 women having hysteroscopic septoplasty whose postoperative management included (1) no treatment, (2) the use of estrogen alone, (3) estrogens and a copper-containing intrauterine device (IUD), or (4) copper-containing IUD alone [32]. There was no reported difference in the rate of postoperative de novo adhesion formation assessed hysteroscopically, and there were no differences in pregnancy outcomes.

Six RCTs have assessed the role of semi-solid (gel) adhesion barriers used postoperatively [33–38] as they may be suitable for preventing IUAs owing to high sensitivity and prolonged time on an injured surface such as the postoperative endometrium [39]. These studies randomized women and compared polyethylene oxide-sodium carboxymethylcellulose gel and hyaluronic acid derivatives against control groups or against each other. For two of these studies, procedures unrelated to pregnancy were examined and reported a significant reduction in de novo adhesion formation when barriers were used compared with controls (3/55 [6%] vs 12/55 [22%] [33] and 7/67 [10%] vs 17/65 [26%] [35]; *p <* .05 for both studies). The third of these studies did not report a reduction in de novo adhesion formation in blinded follow-up with 13/18 (72%) treated women versus 15/22 (68%) women in the control group with no adhesions at 9 weeks follow-up [38]. Adhesions were more likely to be severe in the control group, although not statistically significant. Unfortunately, none of these studies report data on subsequent pregnancy.

The sixth of these RCTs examined primary prevention in women following hysteroscopic removal of retained products of conception and demonstrated no statistical difference for rate of moderate to severe adhesions at 6 to 8 weeks following the procedure (1 woman [4%] receiving barrier vs 3 [14%] controls; *p =* .3) or subsequent pregnancy (7 women [27%] in the barrier group vs 3 [14%] controls *p =* .5) in the 20-month mean follow-up period [34]. The seventh RCT followed 150 women who underwent suction curettage after incomplete, missed, or recurrent miscarriage [36]. Fifty women were randomized to receive an adhesion barrier, and 100 patients served as the control group. In the adhesion barrier group, 32 of 32 patients (100%) became pregnant within 8 months following the procedure compared with 34 of 56 patients (54%) in the control group. Adhesions were found in 1 of 10 women (10%) receiving treatment compared with 7 of 14 (50%) in the control group who had not become pregnant. No adverse events were reported in the treatment group.

In the final RCT studying primary prevention, alginate carboxymethylcellulose hyaluronic acid was compared with carboxycellulose hyaluronic acid in 187 women having various types of hysteroscopic surgery and showed no difference in adhesion severity between the two groups overall, although the alginate carboxymethylcellulose hyaluronic acid was reported to be a better primary prevention product (*p =* .02) [37].

The surgical approach may impact subsequent adhesion formation with retrospective data reporting that a hysteroscopic approach may have benefit over blind curettage [40–42] or ultrasound-guided curettage [43]. Whereas these studies also report an earlier time to next pregnancy, their methodological limitations indicate the need for further evaluation. The type of hysteroscopic procedure being performed may also impact healing and determine subsequent formation of IUAs. Prospective evaluations of hysteroscopic procedures report that the endometrium heals fastest with polypectomy and slowest following septoplasty [44]. The lowest incidence of adhesion formation follows polypectomy with the highest rate of adhesion formation following multiple fibroid resection [45]. The mode of hysteroscopic surgery may be important with avoidance of electrosurgery for myomectomy where adhesions have been documented adjacent to the excised pathology [46–48]. More recently, a large retrospective cohort study has been published reporting lower rates (4%) of IUAs with a hysteroscopic myomectomy technique that combines minimal use of radio frequency electrical energy with cold loop dissection [47].

#### Guidelines for primary prevention of intrauterine adhesions

1. The risk for de novo adhesions during hysteroscopic surgery is impacted by the type of procedure performed with those confined to the endometrium (polypectomy) having the lowest risk and those entering the myometrium or involving opposing surfaces a higher risk. Level B
2. The method of pathology removal may impact the risk of de novo adhesions. The risk appears to be greater when electrosurgery is used in the non-gravid uterus and for blind versus vision-guided removal in the gravid uterus. Level C
3. The application of an adhesion barrier following surgery that may lead to endometrial damage significantly reduces the development of IUAs in the short term, although limited fertility data are available following this intervention. Level A

### Management of intrauterine adhesions

As IUAs are not life-threatening, treatment should be considered only when there are signs or symptoms of pain, infertility, recurrent pregnancy loss, or menstrual abnormalities including hematometra. Surgery has been the criterion standard in the management of Asherman syndrome; however, there are no RCTs comparing surgical intervention and expectant management nor are there RCTs comparing different methods of surgical interventions for Asherman syndrome. The primary objective of any intervention is to restore the normal volume and shape of the endometrial cavity and cervical canal and to facilitate communication between the cavity and both the cervical canal and fallopian tubes. This will allow both normal menstrual flow and adequate sperm transportation for fertilization and implantation to occur.

#### Expectant management

The limited data supporting a role for expectant management, published in 1982, demonstrated resumption of menstruation in as many as 78% within 7 years from diagnosis of IUAs and pregnancy in 45.5% of women [49].

#### Cervical probing

Cervical stenosis without damage to the uterine cavity or endometrium has been treated using cervical probing with or without ultrasound guidance [50]. All available data were accrued before the advent of hysteroscopically directed adhesiolysis, and uterine perforation has been reported after blind cervical probing. Consequently, this technique currently has a limited role.

#### Dilation and curettage

Dilation and curettage was the primary mode of management before the widespread use of hysteroscopy, and reported results included return to normal menses in 1049 of 1250 women (84%), conception in 540 of 1052 women (51%), miscarriages in 142 of 559 pregnancies (25%), term delivery in 306 of 559 pregnancies (55%), premature delivery in 50 of 559 pregnancies (9%), and complicated by placenta accreta in 42 of 559 pregnancies (8%) [49]. The severity of adhesions in this group is unknown, though most were likely mild. With the availability of hysteroscopy, dilation and curettage should not be performed as accurate diagnosis and classification are not possible and further damage to the endometrium may occur.

#### Hysteroscopy

Hysteroscopic treatment enables lysis of IUAs under direct vision and with magnification. The uterine distention required for hysteroscopy may itself lyse mild adhesions, and blunt dissection may be performed using only the tip of the hysteroscope [51]. The more lateral the adhesions and the greater their density, the more difficult the dissection and the greater the risk of complications such as uterine perforation [4, 52]. Monopolar [26, 53–56] and bipolar [57–59] electrosurgical instruments and the Nd-YAG laser [26, 54, 60] have been described as techniques used to lyse adhesions under direct vision, with the advantages of precise cutting and good hemostasis. Disadvantages include potential visceral damage if uterine perforation occurs [8], further endometrial damage predisposing to recurrence of IUAs [61, 62], cost, and the degree of cervical dilation required to accommodate operative instruments. None of these techniques has been compared with any other; consequently, there is no available evidence that one method is superior to any other. Indirect evidence exists to avoid electrosurgery during adhesiolysis owing to the potential risk for further endometrial damage [63]. Mechanical division of adhesions by scissors [7, 26] and needle [64, 65] are described as modes of surgical treatment. Surgical treatment may also take place in an office or outpatient setting with outcomes similar to those in an inpatient setting [66].

#### Other hysteroscopic techniques

Techniques have been described for the treatment of severe cohesive IUAs when typical hysteroscopically directed techniques are not possible or safe. Myometrial scoring has been effective for the creation of a uterine cavity in women with severe IUAs. In this technique, six to eight 4-mm-deep incisions are created in the myometrium using electrosurgery with a Collins knife electrode from the fundus to the cervix. These incisions enable widening of the uterine cavity. Anatomic success has been reported in 71% of patients in one small series [67], and 51.6% in another [53], with pregnancy achieved in 3 of 7 women in the small series (42.9%) and 12 of 31 women in the other (38.7%).

#### Additional guiding techniques for hysteroscopy

Fluoroscopically guided blunt dissection of severe adhesions has been described using a hysteroscopically directed Tuohy needle under image intensifier control with the patient under general anesthesia [64]. This technique is costly, exposes the patient to ionizing radiation, and is technically challenging. Its advantages include use of a narrow hysteroscope, reduced risk of uterine perforation, and reduced risk of visceral damage should perforation occur, because no energy source is applied [65, 68]. A similar technique is described in an ambulatory setting using local anesthesia [69], with described success in mild adhesions only.

Transabdominal ultrasound has been described as a technique to guide hysteroscopic division of IUAs [4, 62, 67, 70, 71]. Advantages of the technique include the availability of ultrasound and its noninvasive nature; however, uterine perforation has been reported in as many as 5% of cases [58, 67, 72]. Laparoscopic guidance is reported to aid hysteroscopically directed division of severe IUAs and enable concurrent inspection of the pelvic organs [58, 67, 72].

Another approach described for treatment of IUAs with cavity obliteration is the use of a cervical dilator sequentially directed from the cervical canal toward the two ostia, creating two lateral landmarks and a central fibrous septum, which is then divided transcervically with a hysteroscopic technique under laparoscopic guidance. A small series of six women has been reported, with uterine perforation in two women and substantial hemorrhage in another [72]. The increased cost and potential morbidity associated with laparoscopy must be considered, and despite improved fertility, with such limited data and high morbidity, this technique cannot be recommended.

### Nonhysteroscopic methods of treating intrauterine adhesions

Laparotomy, hysterotomy, and subsequent blunt dissection through adhesions using a finger or curette have been traditional treatments for severe IUAs [6, 50, 58, 62]. A review of 31 cases and case series treated using this approach reported conception in 16 of 31 women (52%), with live births in 11 (38%) including 8 (26%) who delivered at term. Of the 16 women who conceived, placenta accreta complicated the pregnancy in 5 (31%) [49]. In contemporary practice, this technique is rarely used and is reserved only for severe cases in which other techniques are not practical or possible [73].

#### Guidelines for the surgical management of intrauterine adhesions

1. Hysteroscopic lysis of adhesions by direct vision and a tool for adhesiolysis is the recommended approach for symptomatic IUAs. Level B
2. There is no evidence to support the use of blind cervical probing. Level C.
3. There is no evidence to support the use of blind dilation and curettage. Level C.
4. For women with IUAs who do not wish any intervention but still want to conceive, expectant management may result in subsequent pregnancy; however, the time interval may be prolonged. Level C.
5. Adjunctive interventions to aid adhesiolysis include ultrasound, fluoroscopy, and laparoscopy. There are no data to suggest that these prevent perforation or improve surgical outcomes and are likely dependent on clinical skills and availability. However, when such an approach is used in appropriately selected patients, it may minimize the consequences if perforation occurs. Level B
6. In the presence of extensive or dense adhesions, treatment should be performed by an expert hysteroscopist familiar with at least one of the methods described. Level C.

### Secondary prevention

Having undertaken surgical adhesiolysis, it is recognized that recurrence is common and may occur in 30 to 66% of women treated for IUAs [26, 53, 74–76]. Methods to reduce recurrence have been assessed by an increasing number of randomized trials using a variety of solid and semi-solid (gel) barriers. Traditional solid barrier techniques of separating the uterine walls following adhesiolysis include the use of an IUD, amnion graft, or stent, typically comprising an intrauterine catheter with an inflatable balloon tip. The use of gels such as hyaluronic acid and polyethylene oxide-sodium carboxymethylcellulose has also been subjected to more stringent investigation, and in total, five RCTs are currently evaluating outcomes for secondary prevention strategies.

#### Solid barriers

Insertion of an IUD to separate the endometrial layers after lysis of IUAs has been described for many years [7, 49, 77]. Copper-containing and T-shaped IUDs cannot be recommended because of their inflammation-provoking properties [78] and small surface area [79], respectively. An inert loop IUD (e.g., Lippes loop) is considered the IUD of choice when treating IUAs [4], although it is no longer available in many geographic areas. In a prospective comparative study of 71 women, the use of second-look hysteroscopy was evaluated following insertion of a Lippes loop and estrogen and progestin treatment for 2 months [77]. Women in group 1 underwent early repeat hysteroscopy at 1 week and then reassessment following removal of the IUD at 2 months following index procedure. Group 2 did not have an early repeat hysteroscopy. There was no difference in pregnancy rates or live births and no comparison to women not having an IUD. A randomized study compared an IUD in 80 women with an intrauterine balloon stent in 82 women, each placed for 1 week following hysteroscopic treatment of adhesions [80]. The outcome measure was hysteroscopically rated adhesion score at 1 to 2 months following index treatment, and this study reported no difference in adhesion reformation rate between the balloon group (30%) and the IUD group (35%). This study did not report pregnancy outcomes or compare adhesions in women not receiving any postoperative intervention. In a small nonrandomized study, postoperative IUD plus hormone therapy was compared with hormone therapy alone, with no significant difference reported for adhesion reformation [81]. The risk of infection when an IUD is introduced into the uterus immediately after adhesiolysis is estimated to be 8% [82], and perforation of the uterus during IUD insertion has been reported [82].

The use of a Foley catheter for 3 to 10 days following surgical lysis of IUAs is similarly reported to act as a physical intrauterine barrier [7, 50, 56, 69, 83, 84]. A nonrandomized study compared the use of an inflated pediatric Foley catheter in place for 10 days postoperatively in 59 patients with that of an IUD in situ for 3 months in 51 patients [82]. There were fewer infections in the Foley group and a lower recurrence rate of IUAs as assessed using HSG [82]. Although amenorrhea continued in 19% of women in the Foley group and 38% in the IUD group, the fertility rate was relatively low in both groups: 20 of 59 (34%) and 14 of 51 (28%), respectively. In a study of 25 women with moderate to severe IUAs, use of a fresh amnion graft over an inflated Foley catheter prevented recurrence of IUAs in 52% of women, although follow-up fertility data and complications were not reported [83].

A three-armed pilot RCT assessed fresh amnion versus dried amnion grafts versus intrauterine balloon alone [85]. Forty-five women were randomized (15 in each group), and each underwent diagnostic hysteroscopy 2 to 4 months following treatment. Amnion grafts reduced adhesions significantly more than the balloon alone (*p <* .003), and fresh amnion was superior to dried amnion (*p <* .05). Ten women (23%) conceived with six (60%) having a miscarriage.

The issue of infection with the insertion of an intrauterine stent has been assessed in an RCT of 60 women (30 women randomized to receive the stent; 30 women as a control) [86]. Hysteroscopic procedures were performed, and the outcome measure was bacterial colonization 30 days after the procedure. There was no difference between control (13 and 33%) and stent (10 and 30%) for bacterial colonization rates before and after stent placement suggesting that infection risk is not substantially impacted by the use of an intrauterine stent.

#### Semi-solid barriers

A number of gel adhesion barriers are reported to be successful at reducing the risk of adhesion recurrence after surgical treatment of IUAs [35, 36, 87]. Auto-cross-linked hyaluronic acid gel may be suitable for preventing IUAs because of high sensitivity and prolonged time on an injured surface such as the postoperative endometrium [39]. An RCT of 84 women compared auto-cross-linked hyaluronic acid gel with no therapy after surgical treatment of IUAs. Postoperative ultrasound studies demonstrated that the walls of the uterine cavity remained separated for at least 72 h in the barrier group. At second-look hysteroscopy 3 months after the procedure, IUAs were significantly reduced in women receiving the adhesion barrier compared with the control group (6 of 43 [14%] vs 13 of 41 [32%]; *p <* .05) [87]. Fertility data were not reported in this study.

A retrospective cohort study compared balloon catheter, IUD, hyaluronic gel, and control groups for the reduction of IUAs and found the reduction to be significantly greater in the balloon group compared with the other three groups (*p <* .001). The reduction of IUAs in the IUD group was greater than those of the gel group (*p <* .001) and control groups (*p <* .001), and the reduction in the gel group was not different than the control group [88].

Data from randomized animal studies have reported an increase in pregnancy rate when hyaluronic acid barriers are used following induced IUAs [89]. It remains to be seen if the decrease in adhesion reformation rate extrapolates into increased subsequent pregnancy success following treatment with a gel barrier.

#### Hormonal treatments

Postoperative treatment with estrogen therapy (a daily oral dose of 2.5 mg conjugated equine estrogen with or without opposing progestin for 2 or 3 cycles) [24, 64, 65, 73] has been described after surgical treatment of IUAs. No comparative studies have been performed to investigate dosage, administration, or combination of hormones. One nonrandomized study reported that hormone treatment alone is as effective as hormone treatment and IUD in combination [81].

#### Techniques to increase vascular flow to endometrium

Various studies have described use of medications such as aspirin, nitroglycerine, and sildenafil citrate to increase vascular perfusion to the endometrium [90–93] and enable pregnancy [94]. However, the number of women treated using these therapies remains small, and because all such treatment is off-label, these medications cannot be endorsed outside of rigorous research protocols.

#### Antibiotic therapy

There are no data to support the routine use of antibiotic therapy before, during, or after surgical treatment of IUAs. The American College of Obstetricians and Gynecologists guidelines for antibiotic use in gynecologic procedures do not recommend antibiotic use for diagnostic or operative hysteroscopy [95]. There is, however, a theoretic risk of secondary infection, and it has been proposed that infection may be a primary cause of IUAs. This has led many surgeons to treat women undergoing surgical lysis of IUAs with preoperative or intraoperative antibiotic therapy, and some continue with postoperative antibiotic therapy; however, at this time, there is no evidence to support or refute the use of antibiotic therapy.

### Stem cell treatments for intrauterine adhesions

The use of human stem cell treatments for the reconstruction of the endometrium following substantial damage and IUA formation has been hypothesized for some time [96], with studies from animal models showing substantial promise in this area of medical treatment [97–99]. From the first prospective series in humans, 16 women with substantial hysteroscopically confirmed IUAs were treated by uterine intravascular infusions of BMDSC [100]. Clinical, hysteroscopic, and fertility data are reported subsequently, with menstrual function returning to normal within 6 months of BMDSC infusion and three spontaneous pregnancies and seven pregnancies following in vitro fertilization recorded. These initial data from a human series represent the first adjunct treatment of this type for the treatment of Asherman syndrome with successful menstrual and fertility outcomes. It is imperative that well-conducted RCTs are performed to establish the role of BMDSC treatment in addition to or independent of surgical treatments before it is made available to women.

#### Guidelines for secondary prevention of intrauterine adhesions

1. The use of an IUD, stent, or catheter appears to reduce the rate of postoperative adhesion reformation. There are limited data regarding subsequent fertility outcomes when these barriers are used. Grade A
2. The risk of infection appears to be minimal when a solid barrier is used compared with no treatment. Grade A
3. There is no evidence to support or refute the use of preoperative, intraoperative, or postoperative antibiotic therapy in surgical treatment of IUAs. Grade C
4. If an IUD is used postoperatively, it should be inert and have a large surface area such as a Lippes loop. Intrauterine devices that contain progestin or copper should not be used after surgical division of IUAs. Grade C
5. Semi-solid barriers such as hyaluronic acid and auto-cross-linked hyaluronic acid gel reduce adhesion reformation. At this time, their effect on post-treatment pregnancy rates is unknown. Grade A
6. Following hysteroscopic-directed adhesiolysis, postoperative hormone treatment using estrogen, with or without progestin, may reduce recurrence of IUAs. Grade B
7. The role of medications designed as adjuvants to improve vascular flow to the endometrium has not been established. Consequently, they should not be used outside of rigorous research protocols. Grade C
8. Stem cell treatment may ultimately provide an effective adjuvant approach to the treatment of Asherman syndrome; however, evidence is very limited and this treatment should not be offered outside of rigorous research protocols. Grade C

#### Postoperative assessment

The recurrence rate is as high as one in three women with mild to moderate IUAs [26, 74, 75] and two of three with severe IUAs [53, 76]. Consequently, and regardless of the surgical intervention used, reassessment of the uterine cavity is considered worthwhile, usually after two to three menstrual cycles following surgery [53]. Ambulatory methods include office hysteroscopy and HSG, with recurrence of more than mild IUAs likely requiring anesthetic and division. Early reintervention with assessment a few weeks after hysteroscopy rather than several months has been suggested in randomized [75] and retrospective studies [99, 100] to both assess and treat recurrence.

#### Guidelines for postoperative assessment after treatment of intrauterine adhesions

1. Follow-up assessment of the uterine cavity after treatment of IUAs is recommended, preferably with hysteroscopy. Grade B

### Outcomes

The outcome measures for treating symptomatic IUAs include adhesion scores, menstrual data, pregnancy rates, and clinical outcomes. The available published evidence is principally retrospective, with a few large-scale data sets now available. The best reported fertility outcome is from a single surgeon who reports a live birth in 674/807 (84%) women followed, although the total number of treated women in this analysis is unclear [101]. A retrospective cohort of 683 women with moderate to severe adhesions treated surgically with postoperative adjuvants including one or a combination of IUD, balloon, estrogen, and hyaluronic acid reported a pregnancy rate of 314/475 (66%) with 201/314 (61%) resulting in a live birth [102]. A national referral center in the Netherlands reported menstrual outcomes for 638 consecutively treated women over a 10-year period [68]. The success rate, defined as normal menstruation, was 95%. However, recurrence of IUAs requiring up to three surgical interventions was reported in 27% of women; those with more severe adhesions at baseline were more likely to have a need for subsequent adhesiolysis.

The etiology for the development of IUAs also appears to impact outcome. Women with IUAs associated with uterine artery embolization [103] or uterine compression sutures placed for postpartum hemorrhage appear to have less favorable outcomes than those with adhesions secondary to intrauterine surgical trauma [104, 105]. The use of gel barriers has been the subject of a meta-analysis that notes that fertility outcomes are generally of poor quality [106]. A primary issue is that the RCTs examining this intervention report primarily on reduction of adhesion reformation and not on subsequent pregnancy. These pooled data do not suggest a benefit for any fertility outcome at this time, and it is essential that future studies report these data.

### Recommendations for future research

Since the previous guidelines, there have been an increasing number of RCTs, particularly evaluating methods for primary and secondary prevention. Specific surgical techniques remain untested by RCTs; however, it is recognized that it would be difficult to investigate this aspect of treating IUAs, with surgical variation, protocol development, and adherence and recruitment issues being problematic. Specific future research pathways may include:

1. Methods of diagnosis that may be predictive of outcome. Sonography incorporating contrast agents, 3D reconstruction, Doppler or power flow studies, and MRI techniques may present new pathways for prognosis of treatment and value when counseling women considering treatment.
2. Demonstrating fertility outcomes from RCTs examining primary and secondary prevention techniques. These are of particular importance given the most common presenting problem of IUAs is subfertility.
3. Further study of BMDSC treatment as a medical alternative to surgery, based on initial studies.

It is recognized that a universal classification system would benefit future research studies, although given the current limitations of any single classification system, this is unlikely to occur in the foreseeable future.

## Appendix

This report was developed under the direction of the Practice Committee of the AAGL as a service to its members and other practicing clinicians. The members of the Practice Committee have reported the following financial interest or affiliation with corporations: Jason A. Abbott, FRANZCOG Ph.D.—Consultant: Hologic, Inc., Stryker Endoscopy, Vifor Pharmaceuticals; Malcolm G. Munro, M.D.—Consultant: Aegea Medical, Boston Scientific Corp., Inc., Gynesonics, Hologic, Stock Ownership: Channel Medical; Sony S. Singh, M.D., FRCSC, FACOG—Speakers Bureau: AbbVie, Allergan, Bayer Healthcare Corp.; Stacey Scheib, M.D.—nothing to disclose; Tiffany R. Jackson, M.D.—nothing to disclose; Frank Jansen, M.D., Ph.D.—nothing to disclose; E. Britton Chahine, M.D., FACOG—nothing to disclose.

The members of the AAGL Guideline Development Committee for Intrauterine Adhesions reported financial interest or affiliation with corporations: Jason A. Abbott (Chair), FRANZCOG Ph.D—Consultant: Hologic, Inc., Stryker Endoscopy, Vifor Pharmaceuticals; Malcolm G. Munro, M.D.—Consultant: Aegea Medical, Boston Scientific Corp., Inc., Gynesonics, Hologic, Stock Ownership: Channel Medical; Rebecca Deans, M.D. (nothing to disclose), Mark Hans Emanuel, M.D.—Stock ownership: Smith & Nephew Endoscopy, IQ Medical Ventures; from the European Society of Gynaecological Endoscopy (EGSE): Attilio Di Spiezio Sardo, M.D., Ph.D. (nothing to disclose), Rudi Campo, M.D., Consultant: Karl Storz Endoscopy, Tutlingen, Germany; and Rudy Leon De Wilde, M.D., Ph.D. (Consultant: Nordic Pharma, Karl Storz, Gynesonics).
